# Correction: Avşar Aydın, E. 3D-Printed Graphene-Based Bow-Tie Microstrip Antenna Design and Analysis for Ultra-Wideband Applications. *Polymers* 2021, *13*, 3724

**DOI:** 10.3390/polym16060782

**Published:** 2024-03-12

**Authors:** Emine Avşar Aydın

**Affiliations:** Department of Aerospace Engineering, Adana Alparslan Türkeş Science and Technology University, Adana 01250, Turkey; eaydin@atu.edu.tr

In the original publication [1], there was a mistake in Figure 11, specifically the radiation pattern measured (dotted line) and simulated (straight line) at a frequency of 9.5 GHz (E-plane graphics on the left side and H-plane graphics on the right side). Figure 11 was presented as additional information, and removing it will not affect the paper’s main findings or conclusion. With this correction, the order of some figures has been adjusted accordingly. The author states that the scientific conclusions are unaffected. This correction was approved by the Academic Editor. The original publication has also been updated.
